# Measuring Nursing Home Performance Using Administrative Data

**DOI:** 10.1177/10775587221108247

**Published:** 2022-07-23

**Authors:** Bram Wouterse, Pieter Bakx, Albert Wong

**Affiliations:** 1 Erasmus University Rotterdam, The Netherlands; 2 National Institute for Public Health and the Environment (RIVM), Bilthoven, The Netherlands

**Keywords:** nursing homes, quality of care, long-term care

## Abstract

To improve the quality of nursing home care, reliable estimates of outcomes are essential. Obtaining such estimates requires optimal use of limited data, especially for small homes. We analyze the variation in mortality and hospital admissions across nursing homes in the Netherlands during the years 2010–2013. We use administrative data on all nursing home clients. We apply mixed-effects survival models, empirical Bayes estimation, and machine-learning techniques to optimally use the available longitudinal data. We find large differences in both outcomes across nursing homes, yet the estimates are surrounded by substantial uncertainty. We find no correlation between performance on mortality and avoidable hospital admissions, suggesting that these are related to different aspects of quality. Hence, caution is needed when evaluating the performance of individual nursing homes, especially when the number of outcome indicators is limited.

## Introduction

To improve the quality of nursing home care, good estimates of nursing home performance are essential. Clients who have to choose a nursing home need reliable indicators of nursing home outcomes, and so do providers who want to improve and compare their performance to others, and policymakers who want to improve the quality of care, for instance through pay-for-performance schemes. Yet, obtaining good measures of an individual nursing home’s performance is difficult. Indeed, the World Health Organization and the Organisation for Economic Co-operation and Development (OECD) conclude that “quality measurement in LTC (long-term care) is an important area requiring further policy development . . .” (Barber et al., 2021).

One of the main challenges in identifying nursing home performance is obtaining estimates that are reliable and efficient. In contrast to hospitals, many nursing homes are small, with only a few dozen new residents per year. For these small homes, the quality estimates will be imprecise and are more likely to take on extreme values than estimates for larger homes (Arling et al., 2007). Moreover, the small scale also makes it harder to adjust for case-mix differences. Together, these features mean that the scope for investigating the variation in quality across individual nursing homes is limited. It might be for this reason that, with the exception of Arling et al. (2007), most of the available research does not focus on the variation outcomes across individual homes but instead studies the relation between one observable feature of either the facility or the institutional context and nursing home outcomes. These features include nursing home characteristics (for-profit versus not-for-profit status; Chou, 2002; Grabowski et al., 2013), staffing ratios (Lin, 2014), patient characteristics such as whether the admission is paid for privately or by Medicaid (Grabowski & Gruber, 2007), market characteristics like competition intensity (Jung & Polsky, 2014), and incentives from financing (Werner, Konetzka, & Polsky, 2013), reimbursement rates (Reichert & Stroka, 2018), or public reporting (Konetzka et al., 2013; see Norton, 2016, for a review). Although the impact of specific features on outcomes provides important insights for long-term care policy, these studies necessarily ignore variation within the groups of nursing homes sharing the same characteristic. Hence, they do not estimate the full variation in outcomes among nursing homes needed for informing clients, providers, and policymakers.

### New Contributions

The main research question in this article is

**Research Question 1:** What is the variation in outcomes across Dutch nursing homes?

We focus on two clinical outcome measures: mortality and hospital admissions. Nursing home quality is inherently multidimensional (Castle & Ferguson, 2010; Mor, 2005), and these two outcomes certainly do not capture all, or even what some may believe to be the most important, aspects of quality. However, they still capture *relevant* aspects of nursing home performance that might also be indicative of overall performance. Indeed, mortality has been used in this way, for example, by Bronskill et al. (2009) and Cornell et al. (2019). A relation between these measures and other (input) measures of nursing home quality has been found. For instance, a positive relation between staffing rates (Friedrich & Hackmann, 2021; Lin, 2014) or staff turnover (Antwi & Bowblis, 2018) and mortality has been found. Similarly, Joyce et al. (2018) find lower hospital admissions in homes with dementia special care units.

Nursing home performance may not be consistent across different quality dimensions (Cornell et al., 2019; Fuller et al., 2019; Mor, 2005; Ryskina et al., 2018; Werner, Konetzka, & Kim, 2013). It is therefore relevant to investigate whether performance on mortality and hospital admissions are correlated. The potential complementarity of these outcomes is important for deciding which indicators to include in the performance measurement of providers (Castle & Ferguson, 2010). This is especially the case for countries such as The Netherlands, where, due to a lack of consistently measured outcome measures (Barber et al., 2021), the policy discussions on quality have been mostly based on process and structure indicators.

Our main contribution is that we improve the efficiency of our estimates by using administrative data that are available for all Dutch nursing home residents and by applying statistical techniques that make full use of the longitudinal nature of our data and take differences in the reliability of the quality estimates across nursing homes into account. Specifically, we use data on nursing home use, hospital admissions, causes of death, and an extensive set of background variables including income, prior health care use, and an indicator of independently assessed care need. The most important advantage of this data is that it contains information on all individuals admitted to any nursing home in the Netherlands and that it is consistently measured over multiple years. This allows us to increase the number of clients we can base our estimates on, which is particularly useful for relatively small nursing homes.

An additional advantage of these data is that we do not have to rely on information that is gathered and reported by nursing homes themselves. Self-reported data are often not consistently measured across nursing homes. They are also prone to bias, as providers might be able to influence reported outcomes (Perraillon et al., 2019), and higher quality nursing homes might be better at administering quality data (this is called ascertainment bias, see Roy and Mor (2005).

Even with access to data on all nursing home admission for the full population for several years, the number of cases to base our quality estimates on remains limited for the smaller nursing homes in our sample. To maximize the efficiency of our estimates, we rely on three statistical techniques. First, we use survival models. In contrast to most studies that use a dichotomous outcome measure such as mortality within 30 days after admission, these models use all available longitudinal information over the entire period an individual is living in a nursing home into account. Second, we apply a hierarchical model (a mixed effects survival model), where the nursing-home indicators are modeled as random effects. This method, which is often applied in other settings but (with the exception of Arling et al., 2007) less so for nursing homes, allows us to obtain efficient estimates of nursing-home-specific outcomes, even if the number of residents is small. Third, in a sensitivity analysis, we use machine learning to select the most relevant risk adjusters from a large number of potential candidates in our administrative data.

## Nursing Homes in the Netherlands

There are four types of institutional care for older people in the Netherlands: postacute rehabilitative care, residential care (assisted-living facilities), permanent nursing home care, and palliative care (hospice care). Long-term care providers may provide more than one type of institutional care but usually provide these types of care in separate wards or departments. In this study, we focus on permanent nursing home care. Nursing homes provide around-the-clock support and care to the elderly who cannot live at home any longer because of functional limitations. In all, 5.3% of the Dutch 65+ population lived in an institution in 2014, which is 40% higher than the OECD average of 3.8% (OECD, 2017).

Virtually all nursing home care in the Netherlands is financed through the public long-term care insurance scheme; private out-of-pocket expenditures on long-term care equaled 0.1% in 2013 (Statistics Netherlands, 2017). Users of long-term care pay a co-payment that depends on their household income, wealth, and household composition and never exceeds their financial means. Although co-payments can be substantial for individuals with a high income, they finance only 8% of total nursing home care costs (Bakx et al., 2016). The co-payments do not depend on the type of nursing home care or the choice of the nursing home, which means that the availability of financial resources does not affect the decision to go to a particular provider. Nursing home care covers most costs of daily living, such as housing and meals, and residents do not have to pay separately for these. Individuals who are eligible for nursing home care can, under some conditions, opt to use publicly financed care in a private setting, with for instance more luxurious living arrangements for which they then have to pay themselves. This use of public care in a private setting has become increasingly popular in the last few years but was still very limited during our study period (Hussem et al., 2020).

Eligibility for public long-term care insurance benefits is determined by an independent assessment agency (Centrum Indicatiestelling Zorg—CIZ) using information about the health, well-being, and functional limitations of the applicant as well as the current living conditions. Assessors may base their decision on desk research, a home visit, and consultation of experts, possibly including doctors and also other health care providers who have provided care to the applicant. This agency decides on the types and amounts of care that someone is eligible for, which together make up a care package. There were 10 care packages during the study period, of which care packages 5 through 8 were for nursing home care (see Table 1). Package 5 is specifically for people with dementia.

**Table 1.** The Care Packages Included in the Sample.

Care package	Description
5	Nursing home care with intensive dementia care
6	Nursing home care with intensive personal care and nursing
7	Nursing home care with highly intensive care, with a focus on supervision (often behavioral problems)
8	Nursing home care with highly intensive care, with a focus on personal care and nursing (problems with activities of daily living and cognitive problems)

Providers of nursing home care are nonprofit private entities. They vary widely in size: Some providers consist of a single nursing home, while others are large regional conglomerates with many locations. Providers are reimbursed through care package-specific per diem rates. Care is contracted by regional single payers. Maximum prices per care package are set by the Dutch Health care Authority, and price variation across providers is limited (Barber et al., 2021).

Eligible individuals are free to choose the nursing home they prefer (Bakx et al., 2015). Although overall nursing home capacity in each of the regions is sufficient, individuals who want to go to a specific nursing home of their preference might be confronted with a waiting list. Because individuals are free to choose, providers have some incentive to provide high-quality care (Blank & Eggink, 2001). However, there is no or very limited marketing by nursing homes. Nursing homes may have specialized wards for certain care packages but do not specialize beyond this level.

Like in many countries, the quality of nursing home care is a topic of ongoing public debate in the Netherlands. In 2017, the National Health care Institute put legally binding requirements for the quality of nursing home care into place (ZIN, 2017). This has led to a substantial increase in the budget for nursing home care, mainly aimed to attract additional staff. However, relatively little is known about structural disparities in nursing home care in the Netherlands, especially regarding client outcomes (Bakx et al., 2020). Some quality information is available online in an accessible form, for example, through the website *Zorgkaart Nederland* (Patientenfederatie Nederland, 2021) which reports client experiences on six dimensions: patient-centered care, quality of care, accommodation, whether appointments are met, whether clients are treated well, and whether staff listens to clients and responds appropriately. Furthermore, nursing home quality is monitored by the Health and Youth Care Inspectorate, which reviews a number of process measures, of which the results are published online, as well as the annual report, online patient reviews, and other sources. The Health and Youth Care Inspectorate may advise nursing homes about improvements if the quality is deemed inefficient, or use legal force including the right to close the nursing home if it does not fulfill all criteria (Health and Youth Care Inspectorate, 2021).

## Data

### Data Sources

We use administrative data on nursing home admissions for the full Dutch population from the period 2010–2013. These data come from the Central Administration Office of the Public Long-Term Insurance Scheme that collects these data to charge co-payments to long-term care users. Admissions are reported to the Central Administration Office by nursing homes. The study population consists of all patients in the Netherlands who were admitted to a nursing home in the study period who (a) are at least 65 years old at the time of admission, and (b) who are admitted for permanent nursing home care. We use information on the care package that a patient receives to exclude those admitted for residential care (defined as having a care package 1–4, see Section 3.3), postacute care (care package 9), or palliative care (care package 10).

The dataset contains information on the date of the nursing home admission and the date of discharge. Furthermore, it contains a provider code that we use to identify to which nursing home a person was admitted. This code is pseudonymized to protect the privacy of the nursing home.

We link this information to administrative datasets using a pseudonymized version of the national identification number of the client. These other data contain the main outcome measures. We take data on inpatient hospital admissions from the National Medical Register for the period 2000–2016 that contains the date of admission and the diagnosis according to the Dutch adaptation of the ninth version of the *International Statistical Classification of Diseases and Related Health Problems* (*ICD*) that was used in the Netherlands during the study period.^1^ Furthermore, we use data on mortality (2010–2017) from the Municipal Registry for the full population.

In addition to these outcomes, the data contain background information on the individuals living in a nursing home, including the long-term care eligibility decision (from the Eligibility Assessment Office), outpatient medicine use (from the National Institute for Health care), demographic characteristics such as their date of birth and gender from the mandatory Municipal Registry, and health care paid for through public health insurance (from the centralized data warehouse of the health insurers—Vektis). The insurance claims data provide comprehensive coverage of expenditures: public health insurance is mandatory and, together with public long-term care insurance and the social support act, covered roughly 89% of all spending on medical care and long-term care in 2013 (Statistics Netherlands, 2020).

### Selection of Nursing Homes

**Figure 1.**
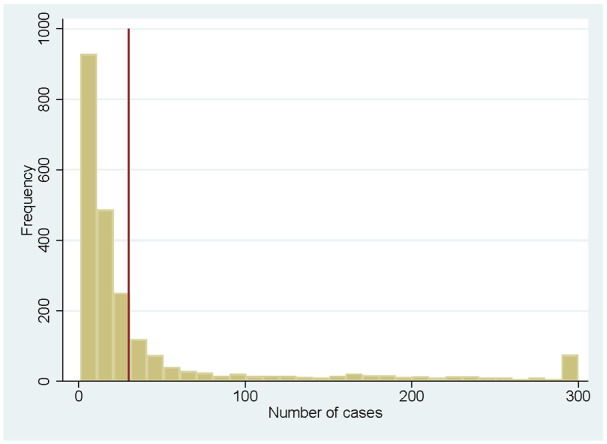
Number of Cases per Provider Code *Note*. Red line indicates the cut-off of 30 cases.

We identify nursing homes based on the provider code. Not all providers use these codes in the same way: In some cases, the code represents one nursing home with one single location serving just a few clients, while in other cases a single code represents multiple locations of the same provider. Because the codes are pseudonymized, we can unfortunately not aggregate all codes to the provider level (or disaggregate to location level). Figure 1 shows the distribution of the number of unique cases (over the whole sample period) across nursing homes. The distribution is highly skewed to the right, with relatively few provider codes having more than 300 newly admitted patients during the study period. The left-hand side of the distribution consists mainly of provider codes with a very low number of admitted clients: the first 25% of provider codes that we observe have only one or two newly admitted clients during our observation period. The median of newly admitted clients is 14. Although we have limited additional information on the characteristics of nursing homes in our data, it seems that some of the provider codes with limited observations are (a) due to actual small-scale facilities with a very limited number of clients, (b) due to administrative reasons (e.g., a nursing home that closes at the start of our observation period), and (c) due to providers that focus on other types of care (e.g., palliative care) but have admitted one or a few patients who officially receive permanent nursing home care.

Because we cannot disentangle the reasons why some provider codes have a limited number of clients, and to obtain reliable estimates, we restrict the sample to provider codes with at least 30 cases during the study period and pool data from four consecutive years. This restriction leaves 624 provider codes (out of 2,317 in total) and 90,519 individuals (out of 119,513) who are newly admitted to one of these homes during the study period. In a sensitivity analysis, we explore the impact of further restricting the sample to homes that have at least 100 cases.

### Outcomes and Confounders

We consider two indicators of nursing home performance: all-cause mortality and hospital admissions. These are severe events that are well-registered in the administrative data. Moreover, both are at least to some extent affected by actions performed by the nursing home staff and other aspects of the environment in the nursing home. In a sensitivity analysis, we compare our outcomes for all-cause mortality to accidents-related mortality, and the outcomes for all-cause hospital admissions to avoidable hospital admissions. We do two things to mitigate case-mix differences. First, we exploit that an independent assessment of the severity of needs is required to receive nursing home care. As mentioned in Section 3.2, we exclude the lowest four care packages that are for residential care and the care packages for palliative care and rehabilitation care. The included care packages are shown in Table 1. The packages give a broad description of the health problems and limitations of the clients, and the types of care that clients are legally entitled to. The eligibility data also include the nature of the health problems (physical, cognitive, or “other”) that was the reason for the eligibility for nursing home care. We include the care package and the nature of the health problem as controls in our analysis. This is expected to filter out a substantial part of the patient heterogeneity.

Second, we control for the following information about the patient in the main analysis: age and gender, year of admission, and health care expenditures in the year prior to admission. In a robustness check, we expand the list of potential case-mix controls by including medicine use in the year prior to admission (2-digit Anatomical Therapeutic Chemical Classification System [ATC] codes), morbidity (measured by the Charlson index based on hospital discharge diagnoses prior to admission; Sundararajan et al., 2004), and health care expenditures prior to admission for 10 types of health care (including general practitioners and hospitals).

### Summary Statistics

Table 2 provides an overview of the main variables used in the analysis, for the total sample of individuals admitted to a nursing home, and for the subsets of individuals who die or are admitted to the hospital during the observation period. Individuals are on average 83 years old when admitted, and 60% are women. Most individuals do not enter the nursing home with the most severe needs: most of the newly admitted residents have care package 5 or 6.^2^ Most admissions are related to physical problems or both physical and cognitive problems. The high prevalence of physical problems is confirmed by other descriptive statistics: Only 13% of the sample did not have any hospital costs in the year prior to admission, while 46% has more than €1,000 of costs. Moreover, a large share uses medication: for instance, 54% of admitted clients use antithrombotic medication, 42% acid-related medication, and 40% antibiotics in the prior year.

**Table 2.** Summary Statistics.

	All		Deceased		Hospitalized	
	*M*	*SD*	*M*	*SD*	*M*	*SD*
Died within 150 days	0.11	0.31	0.19	0.39	0.08	0.28
Hospitalized in 150 days	0.14	0.34	0.10	0.30	0.49	0.50
Avoidable hospitalization in 150 days	0.04	0.20	0.03	0.18	0.16	0.36
Moved in 150 days	0.30	0.46	0.00	0.00	0.17	0.38
Age	82.52	7.20	83.79	7.02	81.37	7.17
Woman	0.60	0.49	0.60	0.49	0.59	0.49
Married	0.39	0.49	0.36	0.48	0.37	0.48
*Care package received*						
5	0.60	0.49	0.66	0.47	0.49	0.50
6	0.33	0.47	0.26	0.44	0.43	0.50
7	0.05	0.22	0.06	0.24	0.05	0.22
8	0.02	0.14	0.02	0.14	0.03	0.17
*Reason for eligibility*						
Cognitive condition	0.52	0.50	0.65	0.48	0.42	0.49
Physical condition	0.75	0.43	0.70	0.46	0.80	0.40
Other	0.12	0.32	0.11	0.32	0.12	0.33
Year of admission						
2011	0.25	0.43	0.27	0.44	0.25	0.44
2012	0.25	0.43	0.23	0.42	0.26	0.44
2013	0.20	0.40	0.19	0.39	0.21	0.41
Hospital admission in prior calendar year	0.26	0.44	0.23	0.42	0.32	0.47
Charlson comorbidity index	0.48	1.01	0.44	0.98	0.64	1.16
*Healthcare expenditures (prior year)*						
ln(healthcare expenditures)	8.12	1.67	8.00	1.84	8.37	1.55
Any hospital expenditures	0.13	0.34	0.15	0.36	0.10	0.30
Hospital expenditures < 1000	0.40	0.49	0.42	0.49	0.37	0.48
Hospital expenditures 1000	0.47	0.50	0.43	0.50	0.54	0.50
*Outpatient medication use (prior year), % by ATC*						
A02: Acid related	0.42	0.49	0.39	0.49	0.46	0.50
A03: Gastrointestinal	0.05	0.23	0.05	0.22	0.06	0.25
A04: Antiemetics	0.00	0.06	0.00	0.06	0.01	0.07
A06: Constipation	0.26	0.44	0.26	0.44	0.28	0.45
B01: Antithrombotic	0.54	0.50	0.53	0.50	0.56	0.50
B03: Antianemic	0.14	0.35	0.15	0.35	0.16	0.37
C01: Cardiac	0.17	0.38	0.18	0.38	0.19	0.39
C03: Diuretics	0.37	0.48	0.37	0.48	0.39	0.49
C07: Beta blockers	0.35	0.48	0.33	0.47	0.38	0.49
C09: Renin-angiotensin	0.38	0.49	0.36	0.48	0.43	0.50
C10: Lipid modifying	0.31	0.46	0.29	0.45	0.34	0.47
D02: Emollients	0.16	0.36	0.16	0.37	0.17	0.37
H02: Corticosteroids	0.13	0.33	0.12	0.33	0.15	0.36
J01: Antibacterials	0.40	0.49	0.40	0.49	0.43	0.50
L01: Antineoplastic agents	0.01	0.11	0.01	0.10	0.01	0.12
L02: Endocrine therapy	0.02	0.15	0.03	0.16	0.02	0.15
M01: Anti-infl/rheum	0.16	0.37	0.14	0.35	0.18	0.38
M04: Antigout	0.03	0.18	0.03	0.17	0.04	0.20
N02: Analgesics	0.20	0.40	0.18	0.39	0.24	0.43
N05: Psycholeptics	0.21	0.41	0.23	0.42	0.20	0.40
N06: Psychoanaleptics	0.26	0.44	0.27	0.44	0.24	0.43
R01: Resp. syst. nasal	0.04	0.20	0.04	0.19	0.05	0.22
R03: Obstr. airway	0.16	0.37	0.16	0.36	0.19	0.39
Observations	93,565		41,867		19,876	

*Note*. ATC = anatomical therapeutic chemical classification system.

**Figure 2:**
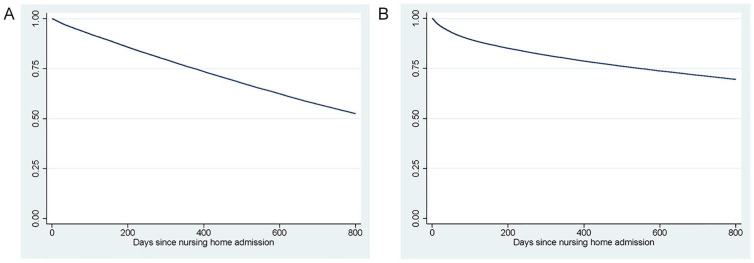
Kaplan–Meier Plots of Death and Hospital Admission: (A) Death and (B) Hospital Admission.

Figure 2 shows the Kaplan–Meier estimates of the survival function for death and hospital admissions for the entire population. In all, 13% of patients die within the first 150 days, and 16% of patients are admitted to the hospital within that time period. A relatively large share of patients leave the nursing home (alive) within the first 150 days: 33 percent (Table 2); these patients often move to another nursing home.

## Method

In our empirical analysis, we have to deal with two specific aspects of the data. The first is that both the outcomes and the exposure to the particular nursing home are longitudinal. The latter is different from medical care where, for example, in the case of hip surgery, the treatment is often a single event after which we can analyze the effects of outcomes over time. Second, our sample partly consists of small nursing homes with relatively few patients, which can lead to inaccurate estimates of nursing-home-specific outcomes.

In Section 4.1, we introduce the mixed effects parametric survival model that we use to deal with the longitudinal nature of the data. In Section 4.2, we then explain how we derive nursing-home-specific quality estimates from this model and deal with the larger uncertainty in these estimates for smaller nursing homes by shrinking these toward the mean. In Section 4.3, we discuss three additional analyses to confirm the robustness of our results: We use different outcome measures, we restrict our sample to large nursing homes, and we use machine learning to expand the set of potential confounders to control for differences in case-mix.

### Survival Analysis Framework

We are interested in the probability of an event (either the first hospital admission or death) for individual i living in nursing home j . We define $T_{i}$ as the time since the nursing home admission at which the event happens for individual i . The outcome in the model is the probability of the event not happening before some moment t . This probability is called the survival: $S(t_{i})=P(T_{i}\geq t_{i})$ .

To estimate the effects of nursing homes on this outcome, researchers often create a binary outcome variable by fixing the time since admission at an arbitrary value $t^{*}$ (e.g., the probability of surviving at least up to 30 days or half a year after admission). The outcome can then be estimated using a linear probability model or a logit or probit model. A disadvantage of this approach is that not all available information is used, as data on the survival function after time $t^{*}$ is disregarded. Instead, we use a survival model approach. This approach uses all available information on the timing of the events by modeling the hazard function λ(t) : the instantaneous rate of the occurrence of the event at any moment t . The hazard rate is defined as $\lambda (t)=\lim_{dt\to 0}((P\{t\leq T<T+dt|T\geq t\})/dt)$ . Survival up to time t is one minus the sum over all hazards faced up to time t : $S(t)=\exp\{-\int_{0}^{t}\lambda (x)dx\}$ .

We use a mixed-effects parametric survival model to estimate the hazard function. In this model, the hazard at a given time t for individual i in nursing home j is a function of fixed effects β relating to the individual’s observed characteristics (in X ) and a nursing home-specific random effect $b_{j}$ :

(1) $$\lambda \left(t_{i,j}\right)=\lambda_{0}\left(t_{i,j}\right)\exp\left(X_{i}\beta +b_{j}\right),$$

with $b_{j}\;:\;N(0,\sigma^{2})$ . Treating the nursing home indicators as random effects, as opposed to a set of fixed effects, is attractive for two reasons. First, there are important efficiency gains, because only one parameter (the random effect variance $\sigma^{2}$ ) is estimated rather than a parameter for each of the nursing homes. Second, as we explain in Section 4.2, this approach helps us to deal with small nursing homes.

The model is a proportional hazard model: the baseline hazard $\lambda_{0}(t)$ is scaled upward or downward based on the individual’s characteristics and nursing home’s performance. The proportional hazard assumption is quite restrictive, but even if it does not fully hold, the estimates provide a “relevant summary of the effect of the covariates over the whole period and a good impression of the overall survival level.” (Van Houwelingen, 2007). Appendix B shows Kaplan–Meier plots of the raw survival and hospital admission risks across subgroups, which provide some graphical support for the assumption. In our setting, the Weibull function provides the best fit for the baseline hazard: $\lambda_{0}(t)=\alpha t^{\alpha -1}$ .

The estimation of the survival functions is complicated by censoring: we do not observe $T_{i}$ for every individual. Censoring may have administrative reasons (the registration of data ends at December 31, 2016) or be caused by competing events, that is, when the patient moves back home or to another nursing home.^3^ Furthermore, death can cause the time to the first hospitalization to be censored (but not vice versa). We rely on the standard assumption that the censoring distribution is independent of the event time distribution so that we can make use of standard models for right-censored data. These models assume that the individuals who are censored can be represented by individuals that still can be observed.

It is not obvious that this assumption holds in the case of hospital admissions. The observations for hospital admissions are censored by the competing event of death. If nursing homes’ effects on mortality in selective (e.g., they only affect the most frail clients) then the estimates for hospital admissions might be biased: at higher values of time since admission the remaining population in nursing homes with high mortality might be healthier and less often admitted to the hospital than in a nursing home with low mortality, where frail clients survive longer. Because we include a relatively large number of covariates on the health of the clients, we expect that the effect of any remaining selective mortality related to unobservable characteristics is limited. However, we cannot fully exclude the possibility that this effect exists, and this is a limitation in the interpretation of our results.^4^

### Estimates of Nursing Home Performance

The estimated parameters from Equation 1 can be used to construct a separate score ( ${\hat{b}}_{j}$ ) for each nursing home j based on the empirical Bayes estimate of $b_{j}$ . This estimate is derived from the likelihood of the actual patient outcomes for nursing home j across all possible values of $b_{j}$ using Bayes’s rule. For this, we use the empirical Bayes mean predictions of the random effects provided by the mestreg command in Stata. The advantage of this estimator is that for (small) nursing homes for which we have relatively little information on the outcomes, the predicted score will be more strongly shrunk toward the mean (i.e., zero).

The estimates of the $b_{j}$ s indicate how a specific nursing home differs from the average nursing home. A positive $b_{j}$ indicates that the adverse outcome is more likely in the nursing home j when compared to the average nursing home, whereas a negative $b_{j}$ indicates that that outcome is less likely. The magnitude of these estimates is difficult to interpret. Therefore, we show the distribution of the nursing home-specific hazard ratio ( $\exp\{{\hat{b}}_{j}\}$ ): the factor by which a nursing home scales up or down an individual’s hazard rate. In addition, we plot the survival at time t for a patient with average characteristics in the median nursing home and the homes in the 10th and 90th percentile.

To explore the properties of these estimates, we perform the following three analyses. First, we compare the correlation between the ${\hat{b}}_{j}$ s for death and for hospital admissions to understand if nursing homes that do well on one characteristic on average also perform well on the other one.^5^

Second, we rank homes based on ${\hat{b}}_{j}$ . To analyze the uncertainty surrounding this ranking, we estimate confidence intervals using a bootstrap procedure. This allows us to assess, for instance, whether we can statistically distinguish nursing homes in the top quartile of the ranking from those in the bottom quartile. Third, we analyze whether nursing homes’ performance is stable over time. To do so, we run separate survival models for (clients admitted in) each calendar year to estimate nursing home- and year-specific effects. We then estimate the correlation of these effects within nursing homes over time.

### Sensitivity Analyses

To assess the sensitivity of the results, we run three sets of additional analyses. First, we use different definitions of the outcome variables: deaths caused by accidents (defined as all deaths with a main cause of death under the accidents chapter of the ICD-9 classification) and avoidable hospital admissions. Avoidable hospital admissions are an important and frequently occurring outcome that has been found to be related to facility practices (Fuller et al., 2019; Grabowski et al., 2008). We define avoidable hospital admissions as admissions for health issues that are known to be preventable by appropriate nursing home care. We base the selection of diagnoses on Carter (2003) and Walker et al. (2009). It includes, among other things, dehydration, kidney/urinary tract infections, injuries form falls, and fractures.

Second, we restrict the sample to the 326 nursing homes with more than 100 cases during the observation period. This restriction allows us to assess to what extent our results might be driven by a lack of power to detect significant effects in small nursing homes.

Third, we make use of the rich linked administrative data to increase the number of potential case-mix controls (see Section 3.3 for an overview). In our main analysis, we base the selection of covariates on what we think, based on theory and empirical literature, are the most relevant predictors of an adverse health outcome within a nursing home. However, our administrative data contain much more detailed information on prior medication use, diagnose-specific hospital admissions, and other potentially relevant characteristics. With this level of detail, it becomes very difficult to decide purely based on a conceptual framework or theory which variables to include. We therefore use a data-driven selection procedure. We apply a two-step procedure. First, we run a linear Lasso regression (Tibshirani, 1996) on the outcomes, which we dichotomize solely for the purpose of variable selection to either dying or being admitted to the hospital within 150 days after admission. From all the potential covariates, the Lasso selects those that jointly are most strongly correlated with the outcome while preventing overfitting by putting a penalty on the sum of the absolute values of the (normalized) coefficients. In the second step, we run the survival models using the variables selected in the first stage as covariates.

## Results

### Main Analysis

#### The Survival Models

The regression coefficients show that women have a smaller hazard of dying or being hospitalized than men (full results in Appendix A). The mortality rate and the avoidable hospitalization rate do not increase consistently with care intensity, but receiving the highest-intensity care (package 8) does increase the chances of both outcomes compared with any of the other packages. Being admitted because of cognitive impairments increases the probability of dying but lowers the probability of avoidable hospital admission.

**Figure 3.**
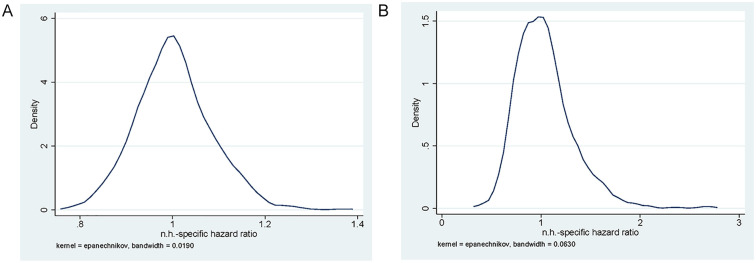
Distribution of Nursing-Home-Specific Random Effects: Empirical Bayes Estimates of the Hazard Ratio: (A) Death and (B) Hospital Admission.

**Figure 4:**
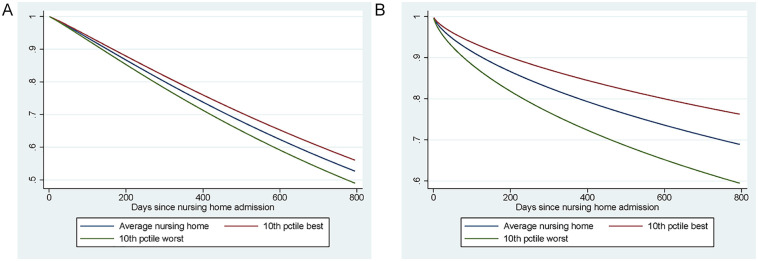
Predicted Survival Function: Baseline Hazard and Hazard in the 10th and 90th Percentile: (A) Death and (B) Hospital Admission.

There is variation in the outcomes among nursing homes, as illustrated by the variation in the nursing-home random effects (Table 3) and the distribution of the empirical Bayes estimates of the nursing-home-specific hazard ratios in Figure 3. For mortality, the hazard ratios range between 0.8 and 1.4 and for hospital admissions between 0.4 and 2.5 (with most of the mass between 0.5 and 1.5). As these hazard rates are hard to interpret, we illustrate what the differences in the outcomes across nursing homes mean by showing how the nursing home-specific effects relate to the predicted outcomes within the first 150 days for the average nursing home patient in Figure 4. In the average nursing home, 90% of the residents are still alive after 150 days. For individuals living in the 10% best-performing nursing homes, this probability is 91% or more, while in the 10% of worst-performing homes it is 89% or less. The mean probability of a hospital admission within 150 days is 11%, while it is 8% or less at the 10% best-performing homes and 15% or more at the worst 10%.

**Table 3:** Overview of the Main Results.

Outcome	Death	Hospitalization	Avoidable hospitalization	Death, accident	Death	Hospitalization	Death	Hospitalization
Sample	full	full	full	full	large homes	large homes	full	full
Selection of covariates							Lasso	Lasso
Random effect variance	0.02	0.11	0.13	0.07	0.01	0.08	0.02	0.09
Event-free survival								
Average, at 150 days	0.90	0.89	0.96	0.99	0.90	0.89	0.90	0.89
10th percentile, at 150 days	0.89	0.85	0.95	0.99	0.89	0.85	0.89	0.85
90th percentile, at 150 days	0.91	0.92	0.97	0.99	0.91	0.92	0.91	0.92
Correlation_t,t−1_	0.28	0.44	0.32	0.15	0.27	0.48	0.27	0.42
Significance (*p* value)	.00	.00	.00	.00	.00	.00	.00	.00
Correlation_death_		−0.05	−0.05	0.42		−0.02	0.00	−0.04
Significance (*p* value)	.00	.20	.19	.00	.00	.72	.00	.33
Number of clients	93,565	93,565	93,565	93,565	76,783	76,783	93,565	93,565
Number of nursing homes	651	651	651	651	328	328	651	651

*Note*. The table reports the estimated variance of the normal distribution of the nursing-home-specific effects, the value of the outcome in the average nursing home and in the 10th and 90th percentile of nursing homes, the correlation between the nursing-home-specific effect between two consecutive years ( $correlation_{t,t-1}$ ), the correlation ( $correlation_{death}$ ) between the nursing-home-specific effect for death and the outcome (if not death), and the number of individual client observations and number of nursing homes. The outcomes are death, hospital admission, avoidable hospital admission, deaths related to accidents. All results pertain to the full sample, with the exception of the analysis for the largest nursing homes ( >100 admissions) only. In all analyses we use the covariates described in Section 3.3, with the exception of Lasso, where we use the Lasso algorithm to select the most relevant predictors.

#### Properties of the Outcome Indicators

**Figure 5.**
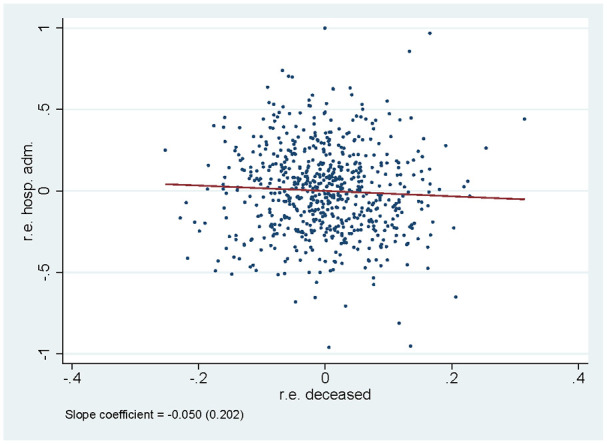
Correlation Between Random Effects for Survival and for Hospital Admissions. Main Specification.

To understand the properties of the two indicators of nursing home performance on outcomes, we consider the correlation between the effects of mortality and avoidable hospital admission, we analyze the uncertainty in the ranking of homes based on the effects, and we look at the correlation of the effects over time. Figure 5 shows a scatter plot of the mortality and avoidable hospital admission effect for each nursing home. There is no significant relationship between the two effects: nursing homes performing well on one indicator, do not necessarily also perform well according to the other one.

**Figure 6.**
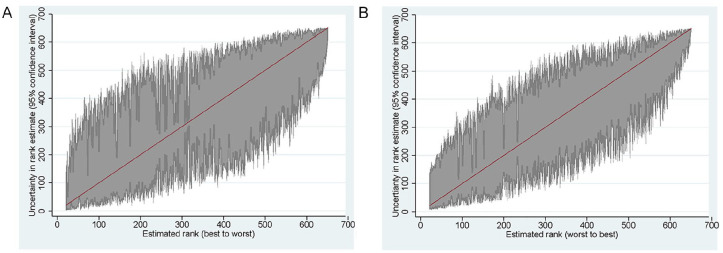
Rankings of Nursing Homes Based on Death and Hospital Admission: Bootstrapped Standard Errors, Based on 500 Runs: (A) Death and (B) Hospital Admission.

Figure 6 shows the ranking of the nursing homes based on their predicted individual effect, from the worst to the best. It also shows the bootstrapped confidence intervals. There is substantial statistical uncertainty around the rankings: for nursing homes in the middle of the distribution, there is a 5% chance that their ranking is at least 200 places higher or lower than estimated. At the same time, we are able to distinguish quite well between the best and the worst-performing homes: for instance, the confidence intervals for the 100 best-performing homes do not overlap with those of the 100 worst-performing ones.

**Figure 7.**
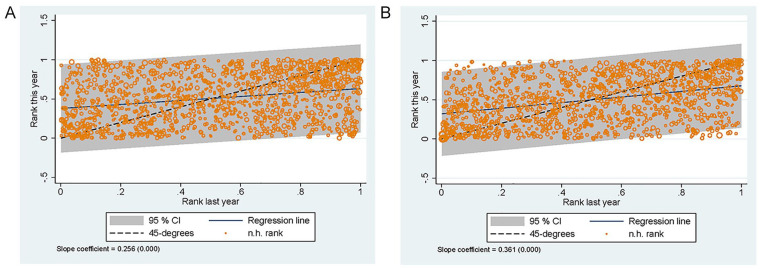
Correlation Between Nursing Home Rank in Year t−1 and Year t for Survival and Hospital Admission: (A) Survival and (B) Hospital Admission.

Finally, we consider the correlation of the effects over time. Our estimates could be affected by random differences in the (unobserved) characteristics of the patients that enter a particular nursing home. If our results would be fully driven by such differences, there would be no correlation between the estimated effects for the same nursing home across different years. To test this, we estimate the models for each year separately, using only patients admitted in each particular year. We then assess the correlation between nursing home ranking in two consecutive years (Figure 7). There is a positive correlation between a nursing home’s current ranking and the ranking in the year before: For mortality, this correlation is 0.256 and for hospital admissions 0.361.

### Sensitivity Analysis

We perform three additional analyses to assess how sensitive our results are to the three main choices we made. Table 3 reports the main outcomes of these analyses. First, we consider two alternative outcomes: accidents-related mortality and avoidable hospital admissions. Relatively few nursing home residents die from accident-related causes: In the average nursing home, a client has a probability of 1% to die from an accident within 150 days after admission, compared to an all-cause mortality of 10%. There is a moderate correlation between the nursing-home-specific effects for all-cause mortality and accident-related mortality (0.42). The intertemporal correlation of the nursing home effects for accident-related deaths is much lower than for all deaths (0.15 instead of 0.28). Avoidable hospital admission is quite frequent within the first 150 days (compared to an overall hospitalization rate of 0.11). There is a strong correlation between nursing-home-specific effects based on all hospital admissions and avoidable hospitalizations (around 70%). Just as with all hospitalizations, the correlation between the effects of avoidable hospitalization and (all-cause) death is not significant. The intertemporal correlation for avoidable hospitalization is somewhat lower than that for all hospitalizations (0.32 instead of 0.44). These findings suggest that, for hospital admissions, the results are not very sensitive to the way in which these are defined. For deaths, we do find differences between all-cause mortality and accident-related mortality, but accident-related mortality does not seem a good alternative because of the relatively few cases and the low correlation between scores over time.

In the second analysis, we limit the sample to the largest nursing homes, that is, those that admitted at least 100 new residents during the study period. The results are very similar to those based on the population of nursing homes with at least 30 cases.

In the third analysis, we expand the number of potential case-mix controls and let the Lasso determine which ones to include. The regression results are in Appendix A Tables 6 and 7. For both outcomes, the algorithm selects many of the variables already included in the main analysis, including age, the care package that someone is entitled to and the reason for eligibility. In addition, a number of indicators for—often aging-related—medicine use are selected. For the regressions with a hospital admission, a large set of disease-specific indicators for prior hospital admissions are included. Surprisingly, however, for both outcomes information on expenditures on specific types of medical care (on top of total spending on medical care) is not selected.

The estimated nursing home-specific effects from the Lasso model have very similar characteristics as those in the main analysis (see Table 3). The distributions of the sets of random effects are also similar and we again find a nonsignificant negative correlation between the effects for mortality and avoidable hospital admissions.

## Conclusion

Information on how individual nursing homes perform is important for policy making, for tying payments to outcomes or for helping clients to choose well-performing providers. However, obtaining good outcome indicators is difficult because of a number of methodological challenges. Many of these challenges are caused or aggravated by the relatively small size of nursing homes and the low number of admissions per year. Hence, making efficient use of the available data is crucial.

We apply three techniques to achieve this efficient use of administrative data on the complete population of Dutch nursing homes. First, we use mixed-effects parametric survival models where nursing home residents are exposed to a nursing home’s quality over an extended period. Second, the empirical Bayes estimates of nursing-home-specific effects that we derive from our models take differences in the sizes of nursing homes into account. Third, we use machine learning to optimally select the most relevant predictors of the outcome variables from an extensive set of administrative data to control for differences in case mix. We analyze the full variation in nursing home mortality and hospitalizations in the Netherlands during the years 2010–2013, but the challenges we address are by no means context-specific.

Our first main finding is that there are differences in both outcomes among Dutch nursing homes. The measurement of these differences is surrounded by a great deal of uncertainty. Still, we can statistically distinguish between poor and high performers. Additional analyses reveal a significant correlation between nursing homes’ outcomes across years, which suggests that variation in outcomes is persistent and not solely caused by random incidental case-mix differences.

Our second main finding is that there is no correlation between the mortality rate and the hospital admission rate of a nursing home’s residents. This might suggest that these two outcomes measure different dimensions of the inherently multidimensional concept of nursing home quality. And, more importantly, the good performance of a nursing home on one of these dimensions is no guarantee for good performance on the other. Future research should try to study this potential selective treatment effect and also consider the correlation with other quality indicators (e.g., process or structure indicators) is equally limited. If this would be the case, it would have important implications for nursing home quality measurement, which then should be based on a broad range of different complementary indicators.

Robustness checks show that these main results are not sensitive to the two most important choices we made, that is, choices with respect to the definitions of the outcomes and to the selection of potential case-mix controls. Moreover, we show that our results are similar when we focus on relatively large nursing homes only.

A first limitation of this study is that we cannot make causal claims: although we can correct for a large set of potentially relevant characteristics and health problems and the scope for selection (e.g., on income) is limited, there could still be unobserved differences at nursing home entry in the condition of patients across nursing homes that might partially explain the variation we observe. Further research may exploit exogenous variation in patients’ nursing home choices (e.g., based on the distance from the place of residence prior to the admissions) to analyze whether these unobserved factors indeed play a role. Similarly, we cannot fully exclude the possibility of selective treatment effects after clients are admitted, which could contribute to the lack of correlation between the observed performance on mortality and hospitalization: If mostly frail clients who suffer from a nursing home’s poor performance on mortality, this could bias the estimate for that home’s performance on hospital admissions, as this estimate would then be more strongly driven by the relatively resilient clients who survive.

A second limitation is that the estimates may be influenced by unobserved differences in beliefs and preferences regarding the effectiveness of hospitalizations and life-prolonging treatment for the oldest old across nursing homes. These differences do not lead to a bias in our estimates: If individuals in a particular nursing home have a relatively high or low probability of hospital admission or death because of the nursing home’s practices regarding end-of-life care, we want our estimates to reflect this. However, it does potentially make the interpretation of outcomes as indicators of nursing home quality more problematic.

A third limitation is that for some nursing homes, we only observe provider codes at the organization level and an organization can have multiple locations. In these cases, the estimates are the average for the organization and may hide variation across individual locations. Furthermore, this limits the ability to compare the estimates at the individual level to other measures of nursing home quality.

A fourth limitation, which likely applies to the study of nursing homes in most countries, is that many homes only treat a limited number of clients. This limits the ability to produce statistically meaningful estimates of the effects of these homes on outcomes. We have had to exclude a large part of the provider codes observed in the data (serving 25 percent of all nursing home clients), as they admitted 30 or less clients during the entire observation period. The empirical Bayes approach allows us to adjust our nursing-home-specific estimates based on the underlying number of observations by shrinking the estimates of smaller homes toward the mean. Although this is in some sense the best approach to make predictions for these homes, it does not overcome the fundamental issue that for small homes there simply is not much information to judge them on.

The small number of newly admitted clients in many nursing homes also means that we faced a trade-off between having enough observations for statistical inference and basing the estimates on the most recent performance of a provider only. To estimate the nursing-home-specific effects, we have used data for 4 years. Although we have found a positive correlation between outcomes across years, an estimate over such a long period is not necessarily indicative of a nursing home’s current performance, which might be the most relevant from a practical perspective. However, restricting the sample to a single year only would mean that the estimates for many of the homes would become very uncertain. Although the trade-off is inescapable, the researcher might extend the framework introduced here to allow for different weights of observation over time, letting more recent observations have a larger influence on the estimated effects.

Our findings raise the question of to what extent nursing home quality can be measured and compared well enough to be used for performance-based payments or for informing prospective residents about relative differences. The clinical relevance of the difference is small when expressed as the difference in the probability of event-free 150-day survival. Furthermore, even when using statistical methods that make efficient use of reliable administrative data for all patients, there is a fair amount of uncertainty about which nursing homes underperform or overperform. The methodological challenges that we have highlighted and addressed in this article are not specific to the indicators that we use or the Dutch context that we analyze. Instead, the challenges are the result of the typical relatively small scale of nursing homes and the fact that the exposure to nursing home quality occurs over an extended period rather than at one point in time. As improving the quality of nursing home care is high on the policy agenda in many countries, finding better ways to address these challenges is important for researchers and policymakers alike.

## Figures and Tables

**Figure B1.**
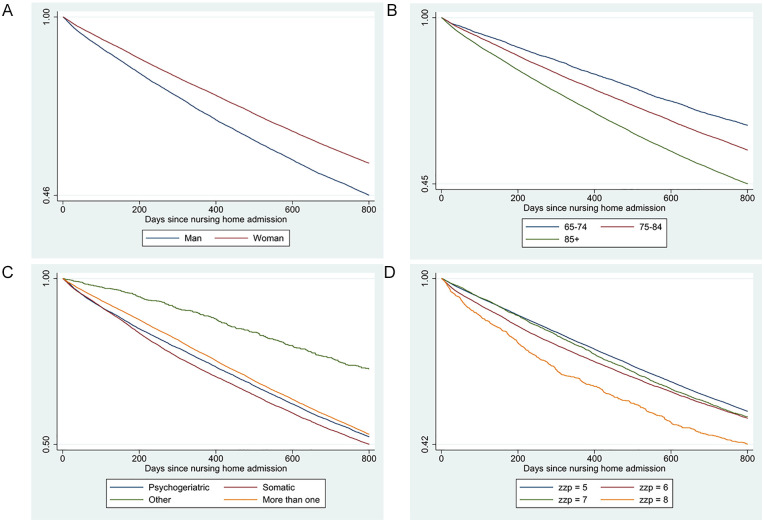
Kaplan–Meier Plots for the Risk of Death Since a Nursing Home Admission, Across Subgroups. (A) By Sex, (B) By Age Group, (C) By Condition, and (D) By Care Package

**Figure B2.**
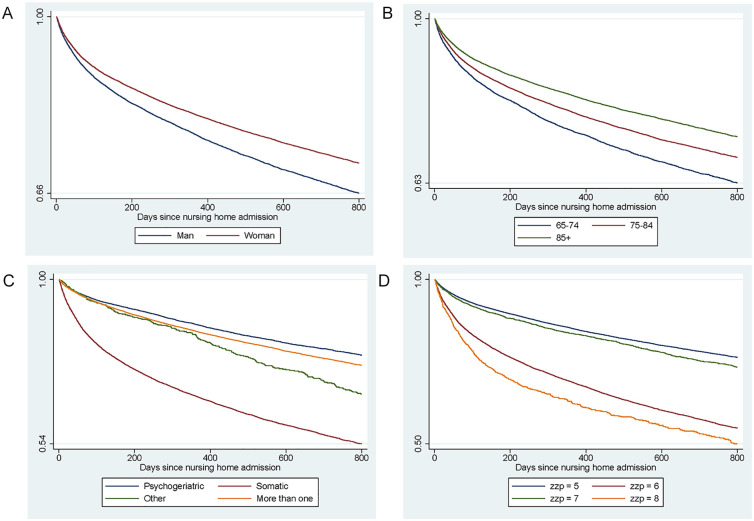
Kaplan–Meier Plots for the Risk of Hospital Admission Since a Nursing Home Admission, Across Subgroups. (A) By Sex, (B) By Age Group, (C) By condition, and (D) By Care Package

## Appendix A

### Regression Tables

**Table A1.** Regression Results for the Mixed Parametric Survival Models. Main Specification.

	Death	Hospital admission
Age	−0.049*** −(0.014)	0.102*** −(0.019)
Age^2^	0.001*** −(0.000)	−0.001*** −(0.000)
Woman	−0.387*** −(0.011)	−0.148*** −(0.014)
Care package 5	−0.589*** −(0.052)	−0.454*** −(0.052)
Care package 6	−0.351*** −(0.048)	−0.205*** −(0.051)
Care package 7	−0.413*** −(0.054)	−0.465*** −(0.061)
ln(health care expenditures)	0.026*** −(0.003)	0.074*** −(0.005)
Reason for eligibility		
Cognitive limitations	0.242*** −(0.020)	−0.622*** −(0.027)
Physical limitations	0.000 −(0.012)	0.0414 −(0.022)
Other limitations	−0.191*** −(0.018)	−0.343*** −(0.025)
Year		
2011	−0.1100*** −(0.014)	−0.040 −(0.022)
2012	−0.233*** −(0.014)	−0.00132 −(0.025)
2013	−0.328*** −(0.016)	−0.057* −(0.028)
Prior hospital admission	−0.087*** −(0.022)	0.092*** −(0.025)
Charlson comorbidity score	0.150*** −(0.010)	0.086*** −(0.010)
Constant	−6.932*** −(0.590)	−8.795*** −(0.774)
ln(α)	0.083*** −0.006	−0.372*** −0.007
var(b)	0.016*** −0.002	0.108*** −0.011
Observations	93,565	93,565

*Note*. Standard errors in parentheses. var(b) is the variance of the nursing-home effects. α is the parameter of the Weibull baseline hazard function.

* *p* < .05. ***p* < .01 . ****p* < .001.

**Table A2:** Regression Results for the Mixed Parametric Survival Models. Lasso, Death.

	Survival
Year in 2011	0.058*** (0.012)
Care package 5	−0.104*** (0.017)
Care package 8	0.279*** (0.048)
Charlson comorbidity score	0.063*** (0.0062)
Married	−0.132*** (0.012)
Age × Woman	−0.003*** (0.000)
Eligible because of cognitive problems	0.231*** (0.018)
*Prior medicine use (ATC group)*:	
B03: Antianemic	0.098*** (0.015)
C01: Cardiac	0.205*** (0.014)
C03: Diuretics	0.159*** (0.012)
H02: Corticosteroids	0.118*** (0.018)
J01: Antibacterials	0.0714*** (0.011)
L02: Endocrine therapy	0.329*** (0.038)
R03: Obstr. airway	0.121*** (0.015)
*Prior hospital use (ISHMT group)*:	
200: Neoplasms	0.515*** (0.035)
1300: Musculoskeletal disease	−0.265*** (0.033)
1900: Injury	−0.088*** (0.016)
Unknown	0.225*** (0.022)
Constant	−7.714*** (0.048)
ln(α)	0.0736*** (0.006)
var(b)	0.018*** (0.002)
Observations	93565

*Note*. Standard errors in parentheses. ATC = Anatomical Therapeutic Chemical Classification System; ISHMT = International Shortlist for Hospital Morbidity Tabulation; var(b) = variance of the nursing-home effects. α = parameter of the Weibull baseline hazard function.

* *p* < .05. ***p* < .01 . ****p* < .001.

**Table A3.** Regression Results for the Mixed Parametric Survival Models. Lasso, Hospital Admissions.

	Hospital admission
Year 2011	−0.007 (0.018)
Care package 5	−0.042 (0.036)
Care package 6	0.167*** (0.040)
Care package 8	0.422*** (0.060)
Eligible because of cognitive problems	−0.497*** (0.026)
Eligible because of physical problems	0.059** (0.022)
Charlson comorbidity score	0.043*** (0.008)
*Prior medicine use (ATC group)*:	
A10: Diabetes	0.122*** (0.019)
B03: Antianemic	0.065** (0.022)
C01: Cardiac	0.076*** (0.020)
C07: Beta blockers	0.077*** (0.015)
C09: Renin-angiotensin	0.148*** (0.017)
H02: Corticosteroids	0.104*** (0.023)
L04: Immunosuppressants	0.220*** (0.054)
N05: Psycholeptics	−0.065*** (0.018)
N06: Psychoanaleptics	−0.027 (0.018)
R01: Resp. syst. nasal	0.161*** (0.039)
R03: Obstr. airway	0.135*** (0.021)
V03: All other	0.471*** (0.076)
*Prior hospital use (ISHMT group)*:	
200: Neoplasms	0.575*** (0.036)
300: Diseases of the blood and bloodforming organs	0.147** (0.055)
700: Diseases of the eye and adnexa	0.379*** (0.041)
900: Diseases of the circulatory system	0.122*** (0.023)
1000: Diseases of the respiratory system	0.214*** (0.036)
1100: Diseases of the digestive system	0.314*** (0.030)
1200: Diseases of the skin	0.305*** (0.058)
1300: Musculoskeletal diseases	0.281*** (0.034)
1400: Diseases of the genitourinary system	0.173*** (0.035)
1900: Injury	0.215*** (0.022)
2100: Factors influencing health status and contact with health services	0.210*** (0.031)
Constant	−5.775*** (0.056)
/ ln(α)	−0.362*** (0.007)
var(b)	0.092*** (0.010)
Observations	93,565

*Note*. Standard errors in parentheses. ATC = Anatomical Therapeutic Chemical Classification System; ISHMT = International Shortlist for Hospital Morbidity Tabulation; var(b) = variance of the nursing-home effects; α = parameter of the Weibull baseline hazard function.

* *p* < .05. ***p* < 0.01 . ****p* < .001.

**Table A4.** Regression Results for the Mixed Parametric Survival Models. Lasso, Death.

	Survival
Year 2011	0.058*** (0.012)
Care package 5	−0.104*** (0.017)
Care package 8	0.279*** (0.048)
Charlson comorbidity score	0.063*** (0.0062)
Married	−0.132*** (0.012)
Age × Woman	−0.003*** (0.000)
Eligible because of cognitive problems	0.231*** (0.018)
*Prior medicine use (ATC group)*:	
B03: Antianemic	0.098*** (0.015)
C01: Cardiac	0.205*** (0.014)
C03: Diuretics	0.159*** (0.012)
H02: Corticosteroids	0.118*** (0.018)
J01: Antibacterials	0.0714*** (0.011)
L02: Endocrine therapy	0.329*** (0.038)
R03: Obstr. airway	0.121*** (0.015)
*Prior hospital use (ISHMT group)*:	
200: Neoplasms	0.515*** (0.035)
1300: Musculoskeletal disease	−0.265*** (0.033)
1900: Injury	−0.088*** (0.016)
Unknown	0.225*** (0.022)
Constant	−7.714*** (0.048)
ln(α)	0.0736*** (0.006)
var(b)	0.018*** (0.002)
Observations	93565

Standard errors in parentheses. ATC = Anatomical Therapeutic Chemical Classification System; ISHMT = International Shortlist for Hospital Morbidity Tabulation; var(b) = variance of the nursing-home effects; α = parameter of the Weibull baseline hazard function.

* *p* < .05. ***p* < .01 . ****p* < .001.

## Notes

1. Although almost all Dutch hospitals participate in this register, coverage is not complete. Results from a sensitivity test that deals with this were very similar to our main analysis (available upon request).

2. When the health of individuals who already live in a nursing home deteriorates, the care package can be upgraded. This does not count as a new case in our analysis.

3. In some cases, we observe two or more consecutive admissions to the same nursing home within short periods of time. If these consecutive admissions occur within 30 days of each other, we treat them as a single admission.

4. Note that selective treatment effects related to unobservables cannot be resolved by standard alternatives used in duration modeling such as using time-varying censoring weights based on observable characteristics (e.g., Willems et al., 2018) or by estimating a competing hazard mode. For a further discussion on selection effects in duration models see Abbring and Van den Berg (2005).

5. We estimate the ex-post correlation between the estimated effects for mortality and hospital admission. An alternative approach would be to estimate both models jointly including the covariance between the random effects. Although this might deliver some efficiency gains, this is also computationally more demanding.
